# Therapeutic potential of an anti-HER2 single chain antibody–DM1 conjugates for the treatment of HER2-positive cancer

**DOI:** 10.1038/sigtrans.2017.15

**Published:** 2017-05-19

**Authors:** Hang Zhang, Yuxi Wang, Yangping Wu, Xiaohua Jiang, Yiran Tao, Yuqin Yao, Yujia Peng, Xiangzheng Chen, Yuyin Fu, Lin Yu, Ruixue Wang, Qinhuai Lai, Weirong Lai, Wenting Li, Yuhuan Kang, Shuli Yi, Ying Lu, Lantu Gou, Min Wu, Jinliang Yang

**Affiliations:** 1 State Key Laboratory of Biotherapy and Cancer Center/Collaborative Innovation Center for Biotherapy, West China Hospital, Sichuan University, Chengdu, China; 2 Chengdu Rongsheng Pharmaceuticals Co., Ltd., Chengdu, China; 3 Research Center for Public Health and Preventive Medicine, West China School of Public, Health/No.4 West China Teaching Hospital, Sichuan University, Chengdu, China; 4 Guangdong Zhongsheng Pharmaceutical Co., Ltd., Dongguan, China; 5 Department of Liver Surgery and Liver Transplantation Center, West China Hospital, Sichuan University, Chengdu, China; 6 Department of Biomedical Sciences, University of North Dakota, School of Medicine and Health Sciences, Grand Forks, North Dakota, USA

## Abstract

Antibody–drug conjugates (ADCs) take the advantage of monoclonal antibodies to selectively deliver highly potent cytotoxic drugs to tumor cells, which have become a powerful measure for cancer treatment in recent years. To develop a more effective therapy for human epidermal growth factor receptor 2 (HER2)-positive cancer, we explored a novel ADCs composed of anti-HER2 scFv–HSA fusion antibodies conjugates with a potent cytotoxic drug DM1. The resulting ADCs, T-SA1–DM1 and T-SA2–DM1 (drug-to-antibody ratio in the range of 3.2–3.5) displayed efficient inhibition in the growth of HER2-positive tumor cell lines and the half-maximal inhibitory concentration on SKBR-3 and SKOV3 cells were both at the nanomolar levels *in vitro*. In HER2-positive human ovarian cancer xenograft models, T-SA1–DM1 and T-SA2–DM1 also showed remarkable antitumor activity. Importantly, three out of six mice exhibited complete remission without regrowth in the high-dose group of T-SA1–DM1. On the basis of the analysis of luminescence imaging, anti-HER2 scFv–HSA fusion antibodies, especially T-SA1, showed strong and rapid tumor tissue penetrability and distribution compared with trastuzumab. Collectively, the novel type of ADCs is effective and selective targeting to HER2-positive cancer, and may be a promising antitumor drug candidate for further studies.

## Introduction

Antibody–drug conjugates (ADCs), combining an antibody with high cytotoxic small-molecule compounds via a linker, are a new class of highly potent anti-cancer drugs. They have been applied for clinical therapy and showed great promise in recent years.^1–3^ ADCs integrate specific targeting capability of the antibody with high cytotoxicity of the small-molecule compounds, leading to significant improvement of drug efficacy while reducing the side effects.^4,5^ Thus far, there are 2 ADCs (Adcetris and Kadcyla) that have been approved by the Food and Drug Interactions,^6,7^ and over 40 ADCs are currently under clinical evaluations.

Human epidermal growth factor receptor 2 (HER2) is an 185 kDa transmembrane receptor-like protein with tyrosine kinase activity.^8,9^ Although with unknown natural ligand, HER2 can be stimulated by interaction with other family members activated by their ligands and then plays important roles in the process of cell proliferation, differentiation, migration as well as anti-apoptosis.^10^ Because of its high expression in a variety of human cancers (for example, breast cancer, gastric cancer, ovarian cancer, non-small-cell lung cancer and others), HER2 has received great attention in anti-cancer research during the past two decades.^11–16^ Several antibody drugs and small-molecule inhibitors have been approved by the Food and Drug Interactions for HER2-positive cancer therapy. Antibody drugs include trastuzumab (Herceptin, Genentech),^17–19^ pertuzumab (Perjeta, Genentech)^20,21^ as well as T-DM1 (Kadcyla, Genentech).^22,23^ In addition, there are two classes of small-molecule tyrosine kinase inhibitors targeting HER2, including lapatinib (Tykerb, Novartis)^24–26^ and afatinib (Giotrif, Boehringer Ingelheim).^27^

T-DM1, the conjugates of maytansine derivative DM1 linked to trastuzumab via bifunctional linker SMCC developed by Genentech, is effective in patients with HER2-overexpressing tumors, including some of those who are resistant to trastuzumab itself.^28–31^ Maytansine can inhibit tubulin aggregation via binding with the periwinkle loci on tubulin as a result of promoting apoptosis of tumor cells.^32^ In a phase III clinical trial, overall objective response rate was 43.6% for patients in the T-DM1 group, and median progression-free survival was 6.4 months, which extended by 3.2 months compared with conventional chemotherapy.^33^ Another ADC, namely, SGN-35, was produced by conjugating anti-human CD30 monoclonal antibody cAC10 with a microtubule inhibitor MMAE. SGN-35 produced a fairly high objective response rate in treating relapsed or refractory Hodgkin’s lymphoma and relapsed or refractory systemic anaplastic large cell lymphoma.^34–36^ The median progression-free survival in the SGN-35 arm was extended by 18.8 months compared with the placebo group.^37^ However, intact antibodies generally have inadequate tissue penetrability and diffusivity, which limit the therapeutic efficacy of the corresponding ADCs for solid tumors. Engineered antibodies featuring similar binding activity but stronger tissue penetrability, such as Fab, scFv (single-chain variable fragment) and diabody, may be utilized as preferred targeting elements for novel ADCs to increase their therapeutic efficacy.

Compared with the intact antibody, scFv not only has similar binding activity but also has features such as being smaller in size, stronger blood vessels and tissues penetrability and lower immunogenicity.^38–41^ We therefore hypothesized that scFv used as a targeting moiety for delivery of drug may have better therapeutic efficacy against solid tumors. However, scFv has a short half-life in plasma, and this becomes a barrier for its clinical application.^42^ Many engineering approaches have been designed to improve the stability of scFv in plasma, and these approaches are also effective in increasing therapeutic accumulation in tumor sites. Human serum albumin (HSA) is one of the major plasma proteins and an ideal drug delivery carrier.^43–47^ Meanwhile, HSA is ready to accumulate in malignant and inflammatory tissues, which are lack of lymphatic drainage, indicating that HSA has a certain feature of tumor targeting.^48^ Consisting of scFv–HSA fusion antibodies as guiding molecules, the half-life of ADCs can be increased and the *in vivo* tumor targeting can be enhanced to a certain extent. This will be a new direction in the development of a new generation of ADCs.

In this study, we prepared two types of scFv–HSA fusion antibodies targeting HER2 by transient expression in mammalian cells. Their binding activity, affinity as well as internalization *in vitro* were investigated. Then, we generated two novel ADCs by conjugating DM1 to anti-HER2 scFv–HSA fusion antibodies and evaluated their *in vitro* and *in vivo* antitumor activities. Furthermore, the distribution and penetrability of the anti-HER2 scFv–HSA fusion antibodies in tumor and normal tissues compared with the intact antibody were analyzed. The results showed that the anti-HER2 scFv–HSA–DM1 was effective and selective for HER2-positive cancer and may be a promising antitumor drug candidate.

## Materials and methods

### Cell lines and culture conditions

HER2-negative human breast cancer cell lines MCF-7 and MDA-MB-231, HER2-positive human breast cancer cell lines SKBR-3, BT474 and ZR-75-1, and HER2-positive human ovarian cancer cell line SKOV3 were obtained from American Type Culture Collection (Manassas, VA, USA). The cell lines were cultured in high-glucose Dulbecco’s modified Eagle medium or Roswell Park Memorial Institute 1640 medium supplemented with 10% fetal bovine serum (Gibco, Life Technologies, Carlsbad, CA, USA), penicillin (100 U ml^−1^) and streptomycin (100 μg ml^−1^) at 37 °C in 5% CO_2_. FreeStyle 293-F cell line was obtained from Life Technologies and expanded in FreeStyle 293 Expression Medium (Gibco).

### Preparation and characterization of anti-HER2 scFv–HSA fusion antibodies (T-SA1 and T-SA2)

T-SA1 and T-SA2 were produced in the FreeStyle 293-F mammalian cell transient expression system that was transfected with the plasmids pTT5/T-SA1 or pTT5/T-SA2 containing complementary DNA (cDNA) of T-SA1 and T-SA2 proteins, respectively. T-SA1 and T-SA2 contained cDNA of an anti-HER2 scFv designed according to amino-acid sequences of V_H_ and V_L_ regions of trastuzumab with a flexible linker and HSA. cDNA was synthesized by Genscript Biotechnology Company (Nanjing, China). Five days after transfection, expression supernatant was collected and proteins purification was performed in two steps by HiTrap protein L affinity chromatography and Superdex 200 Increase gel filtration chromatography (GE Healthcare, Pittsburgh, PA, USA).

The desired proteins were analyzed by 10% SDS-polyacrylamide gel electrophoresis (SDS-PAGE) under natural and denaturing conditions. The loading quantity of each sample was kept consistent. The gel was stained with Coomassie Brilliant Blue G-250 (Bio-Rad, Hercules, CA, USA) and image was scanned using the Bio-Rad Gel Doc 2000 imaging system.

The association constant, dissociation constant and affinity of the complexes consisting of anti-HER2 scFv and recombinant extracellular domain of the HER2 receptor p185^HER2-ECD^ (Sino Biological, Inc., Beijing, China) were determined by BIAcoreX100 (GE Healthcare) bio-molecular interaction analyzer based on surface plasmon resonance.^49^

### Flow cytometric analysis of scFv–HSA fusion antibodies

Breast cancer cell lines MCF-7, SKBR-3 and BT474, and human ovarian cancer cell line SKOV3 were trypsinized. Cells (1×10^6^) were centrifuged, washed with phosphate-buffered saline (PBS) and resuspended in 100 μl PBS (pH 7.2) or PBS containing trastuzumab, T-SA1 or T-SA2 labeled with fluorescein isothiocyanate (FITC) at the concentration of 10 μg ml^−1^ at 4 °C for 30 min, respectively. After incubation, cells were washed three times and resuspended in 500 μl PBS. The fluorescence intensity of FITC was determined using flow cytometer (FACSCalibur, BD, San Jose, CA, USA). Three independent experiments of flow cytometric analysis were conducted.

### Internalization analysis of scFv–HSA fusion antibodies

Breast cancer cell lines MCF-7, SKBR-3 and BT474, and human ovarian cancer cell line SKOV3 were trypsinized. Cells (1×10^6^) were centrifuged, washed with PBS and incubated with T-SA1 or T-SA2 at the concentration of 10 μg ml^−1^ at 4 °C for 30 min, respectively. Then, cells were washed three times. The control of each treatment group labeled with Albumin Antibody-FITC conjugate (Thermo Scientific, Waltham, MA, USA) at 4 °C for 30 min then washed. Others (experimental groups) were resuspended in 100 μl PBS and incubated at 37 °C for 1, 4, 6 or 8 h, respectively. After incubation, cells were centrifuged and labeled with Albumin Antibody-FITC conjugate at 4 °C for 30 min. Cells were washed three times and resuspended in 500 μl PBS. The fluorescence intensity of FITC was determined using flow cytometer (Novocyte, ACEA Bioscience, San Diego, CA, USA). The following formula was used to calculate the internalization efficiency rate of each agent in cells: internalization efficiency rate (%)=[(fluorescence intensity of the control group−fluorescence intensity of the experimental group)/ fluorescence intensity of the control group]×100%.^50,51^

To analyze the visualizing distribution of fusion antibodies in cells, each type of breast cancer cell lines (that is, SKBR-3, BT474 and MCF-7) was divided into six parts and plated onto cover slips in six-well plates and then cultured at 37 °C. After 24 h of culture, two wells of each cell type were incubated with trastuzumab, T-SA1 and T-SA2 labeled with FITC in the concentration of 20 μg ml^−1^ at 4 °C for 30 min, respectively. Then, cells were washed with PBS for three times. For control cells, lysosomes were labeled with Lyso-Tracker Red (Beyotime Biotechnology, Shanghai, China). Next, cells were washed with PBS for three times, and fixed in 4% (W/V) paraformaldehyde at room temperature for 10 min. After washing with PBS for three times, the cell nuclei were labeled with 4,6-diamidino-2-phenylindoledihydrochloride (Sigma-Aldrich, St Louis, MO, USA) in the concentration of 1.5 μg ml^−1^ at room temperature for 5 min. Experimental cells were incubated with culture medium without serum and antibiotics at 37 °C for 6 h. Then, lysosome dying, cell fixation and nucleus staining were carried out according to the above methods. After extensive wash, visualization of immunofluorescence was observed with fluorescence microscope (Olympus, Tokyo, Japan; TH4–200).^52–54^

### Preparation of ADCs

T-SA1–DM1 and T-SA2–DM1 were two kinds of ADCs by conjugating cytotoxic agent DM1 with scFv–HSA fusion antibodies (T-SA1 and T-SA2) via the free amino group of lysine residues. The scFv–HSA fusion antibodies and DM1–MCC were mixed in a molar ratio of 1:7.5 in conjugation buffer (50 mm potassium phosphate, 50 mm sodium chloride, 2 mm EDTA, pH 7.2) and stirred at 25 °C overnight. After centrifuged at 17 000 *g* for 5 min, the supernatant was purified to yield T-SA1–DM1 and T-SA2–DM1 conjugates and replaced to storage buffer (50 mm sodium phosphate, 50 mm sodium chloride, pH 7.2) by a size-exclusion chromatography (Thermo Pierce Zeba Spin Desalting Columns and Devices (Thermo Fisher Scientific, Waltham, MA, USA), 7 K molecular weight cut off). Absorbance of anti-HER2 scFv–HSA–DM1 conjugates was tested at 280 and 252 nm, respectively, and methods for testing drug-to-antibody ratios (DARs) of T-SA1–DM1 and T-SA2–DM1 were established basing on differential ultraviolet spectrophotometry.

### Analysis of DAR

ScFv–HSA fusion antibodies absorb strongly at 280 nm but DM1 absorbs strongly at 252 nm. According to the Lambert Beer’s absorption law (*A*=*εBC*), the formulas to calculate total absorbance of anti-HER2 scFv–HSA–DM1 conjugates at 280 and 252 nm are following:

$$A_{280\;\mathrm{anti}-\mathrm{HER2}\;\mathrm{scFv}-\mathrm{HSA}-\mathrm{DM1}}=\varepsilon_{280\;\mathrm{anti}-\mathrm{HER2}\;\mathrm{scFv}}{\mathrm{BC}}_{\mathrm{anti}-\mathrm{HER2}\;\mathrm{scFv}}+\varepsilon_{280\;\mathrm{DM1}}{\mathrm{BC}}_{\mathrm{DM1}}$$

$$A_{252\;\mathrm{anti}-\mathrm{HER2}\;\mathrm{scFv}-\mathrm{HSA}-\mathrm{DM1}}=\varepsilon_{252\;\mathrm{anti}-\mathrm{HER2}\;\mathrm{scFv}}{\mathrm{BC}}_{\mathrm{anti}-\mathrm{HER2}\;\mathrm{scFv}}+\varepsilon_{252\;\mathrm{DM1}}{\mathrm{BC}}_{\mathrm{DM1}}$$

The extinction coefficients of each component at these wavelengths as follows: *ε*_280 T-SA1_=84 545 m^−1^ cm^−1^, *ε*_280 T-SA2_=134 645 m^−1^ cm^−1^, *ε*_280 DM1_=5700 m^−1^ cm^−1^, *ε*_252 T-SA1_=39 736.15 m^−1^ cm^−1^, *ε*_252 T-SA2_=57 897.35 m^−1^ cm^−1^, *ε*_252 DM1_=26 790 m^−1^ cm^−1^. On the basis of the absorbance at 280 and 252 nm, the average molar concentrations of scFv–HSA fusion antibodies and DM1 can be calculated. All the tests were taken using PerkinElmer (Boston, MA, USA), Lambda 35, UV/VIS ultraviolet spectrophotometer. The instrument was blanked with storage buffer and samples were diluted to proper concentrations. Three independent measurements were conducted and averages were calculated.

$$T-\mathrm{SA1}-\mathrm{DM1}\;\mathrm{conjugates}:\;C_{\mathrm{DM1}}=\left(A_{252}-0.47\times A_{280}\right)/24111C_{T-\mathrm{SA1}}=\left(4.7\times A_{280}-A_{252}\right)/357625.35\mathrm{DAR}=C_{\mathrm{DM1}}/C_{T-\mathrm{SA1}}$$

$$T-\mathrm{SA2}-\mathrm{DM1}\;\mathrm{conjugates}:\;C_{\mathrm{DM1}}=\left(A_{252}-0.43\times A_{280}\right)/24339C_{T-\mathrm{SA2}}=\left(4.7\times A_{280}-A_{252}\right)/574934.15\mathrm{DAR}=C_{\mathrm{DM1}}/C_{T-\mathrm{SA2}}$$

### Characterization of anti-HER2 scFv–HSA–DM1 conjugates

The integrity and purity of anti-HER2 scFv–HSA–DM1 conjugates were verified by SDS-PAGE. Protein concentration was measured based on the method mentioned in the previous section and the loading quantity of each sample was kept consistent. T-SA1, T-SA1–DM1, T-SA2 and T-SA2–DM1 were separated on 10% polyacrylamide gels under natural and denaturing conditions. The gels were stained with Coomassie Brilliant Blue G-250 (Bio-Rad) and images were scanned.

The binding activity comparing anti-HER2 scFv–HSA–DM1 conjugates with their unconjugated antibodies was studied by flow cytometry on HER2-positive cell lines (SKOV3, BT474, SKBR-3 and ZR-75-1) and HER2-negative cell line MCF-7. Trypsinized cells (1×10^6^) were centrifuged, washed with PBS and resuspended in 100 μl PBS (pH 7.2) or PBS containing T-SA1, T-SA1–DM1, T-SA2 or T-SA2–DM1 at the concentration of 10 μg ml^−1^ at 4 °C for 30 min, respectively. Cells were washed three times with PBS and then labeled with Albumin Antibody-FITC conjugate (Thermo Scientific) at 4 °C for 30 min. After incubation, cells were washed three times and resuspended in 500 μl PBS. The fluorescence intensity of FITC was determined using flow cytometer (Novocyte, ACEA Bioscience). Three independent experiments of flow cytometric analysis were conducted. Furthermore, the affinity of two anti-HER2 scFv–HSA–DM1 conjugates, T-SA1–DM1 and T-SA2–DM1, with the extracellular domain of HER2 was analyzed by surface plasmon resonance technology.^55,56^

### *In vitro* cytotoxicity assay

The anti-proliferation activity of scFv–HSA fusion antibodies and their conjugates on breast cancer cells (MCF-7, MDA-MB-231 and SKBR-3) and ovarian cancer cells (SKOV3) were determined by Cell Counting Kit-8 assay (Dojindo Laboratories, Kumamoto, Japan). Cells (1.5–6×10^3^ per well, changes according to the growth rate of different cells) were seeded in 96-well plates with the volume of 100 μl medium for each well. After 24 h of culture, cells were treated with scFv–HSA fusion antibodies or their conjugates of serial dilutions in culture medium. Each drug concentration was plated in triplicate. After 72 h of continuous drug exposure, 20 μl Cell Counting Kit-8 solution was added to each well, and cells were incubated for another 1–3 h. The absorbance at 450 nm was measured by microplate reader (Thermo, Multiskcan MK3). All experiments were measured independently in triplicate. The following formula was used to calculate the cell growth inhibition rate of each agent on cells: cell growth inhibition rate (%)=[(*A*_450_ of control−*A*_450_ of treated cells)/*A*_450_ of control]×100%. The half-maximal inhibitory concentration (IC_50_) value of each agent was statistically analyzed by GraphPad Prism 6.0 software (GraphPad Software, Inc., San Diego, CA, USA).^39,57,58^

### *In vivo* antitumor activity studies

Animal experiments were approved by the Institutional Animal Care and Treatment Committee of the State Key Laboratory of Biotherapy in Sichuan University. Female Balb/c nude mice (age, 4–5 weeks) purchased from (Beijing HFK Bioscience Co., Ltd., Beijing, China) were acclimated for 1 week before the experiment. The SKOV3 ovarian cancer xenograft model was employed to evaluate the *in vivo* activity of anti-HER2 scFv–HSA–DM1 conjugates comparing with their unconjugated antibodies. Mice were given a single point of subcutaneous injection with SKOV3 cells suspension (1×10^7^ cells in 100 μl cell culture medium without serum and antibiotics). When the volume of subcutaneous xenografts reached 200 mm^3^, the mice were divided into five groups (*n*=6). The scFv–HSA fusion antibodies (20 mg kg^−1^), their DM1 conjugates (5, 10 and 20 mg kg^−1^) and control (storage buffer) were administered via tail vein injection to mice on days 0, 4 and 8. Tumor volume and body weight were monitored twice a week. When the volume of subcutaneous xenografts was >1500 mm^3^, the mice were killed.

### *In vivo* distribution of scFv–HSA fusion antibodies

Human ovarian cancer SKOV3 xenograft model was established as mentioned in the previous section. When the volume of subcutaneous xenografts reached 300 mm^3^, these mice were divided into four groups (*n*=4). One nanomolar of trastuzumab, T-SA1 or T-SA2 labeled with Cy5.5 was administered via tail vein injection to mice. At 2, 6, 12 and 24 h after injection, the distribution of trastuzumab, T-SA1 and T-SA2 *in vivo* was observed by the luminescence imaging system. Furthermore, tumor specimens at each time point and normal tissues at 12 h after injection were collected. All tissues were embedded with optimum cutting temperature compound immediately. Then, 5 μm frozen sections from the optimal cross-sectional surface were prepared and cell nuclei were stained with 4,6-diamidino-2-phenylindole dihydrochloride. Frozen sections were observed with laser scanning confocal microscope (Leica, Leica Microsystems, Wetzlar, Germany, DM6000CS) to evaluate the *in vivo* distribution of scFv–HSA fusion antibodies compared with the intact antibody trastuzumab.

## Results

### Construction, expression and characterization of anti-HER2 scFv–HSA fusion antibodies

**Figure 1:**
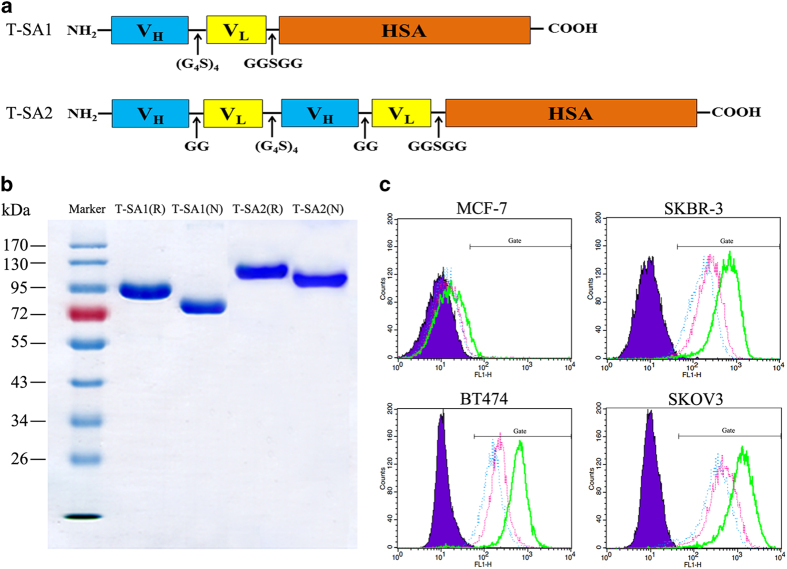
Preparation and characterization of T-SA1 and T-SA2. (**a**) Schematic diagram of T-SA1 and T-SA2 containing anti-HER2 scFv, the linkers and HSA. (**b**) SDS-PAGE analysis of purified T-SA1 and T-SA2. T-SA1 and T-SA2 loaded with reducing (R) and non-reducing loading buffer (N) were separated on a 10% polyacrylamide gel. Marker: PageRuler PlusPrestained-Protein Ladder. (**c**) Binding activity analysis of T-SA1 and T-SA2 in HER2-negative and -positive cells by flow cytometry. The results indicated that T-SA1 and T-SA2 could specifically bind to HER2-positive cells. Blue violet: control group; green: anti-HER2 monoclonal antibody trastuzumab; pink: T-SA1; blue: T-SA2.

A scFv fused with HSA via linker (GGSGG) formed a single-chain T-SA1 (Figure 1a). This scFv was composed of amino-acid sequences of V_H_ and V_L_ regions of anti-HER2 monoclonal antibody trastuzumab connected by a flexible linker (G_4_S)_4_. T-SA2 was composed of two tandem trastuzumab scFvs connected by linker (G_4_S)_4_ fusing with HSA via linker (GGSGG). The V_H_ and V_L_ regions of scFv were connected by linker (GG; Figure 1a). Anti-HER2 scFv–HSA fusion antibodies were transiently expressed in FreeStyle 293-F cells, and purified by protein L affinity chromatography and Superdex 200 Increase gel filtration chromatography. Anti-HER2 scFv–HSA fusion antibodies migrated on 10% SDS-PAGE at the expected molecular weight of 92.9 (T-SA1) and 117.9 kDa (T-SA2) (Figure 1b).

The binding affinity of T-SA1 with recombinant extracellular domain (ECD) of HER2 was 5.910×10^−11^ m by surface plasmon resonance analysis, whereas that of T-SA2 was 1.300×10^−10^ m. The association rate constants of the two antibodies were both at 10^5^ m^−1^s^−1^ level, which was at the same level as trastuzumab. This result indicated that both T-SA1 and T-SA2 have high affinity for HER2 receptor. Furthermore, the binding activity of T-SA1 and T-SA2 with HER2-positive cells was investigated by flow cytometry. As shown in Figure 1c, though slightly inferior shift extent compared with trastuzumab, T-SA1 and T-SA2 could specifically bind to HER2-positive cells instead of MCF-7 cell with low expression of HER2, indicating that anti-HER2 scFv–HSA fusion antibodies have good binding activity to HER2-positive tumor cells.

### Internalization of scFv–HSA fusion antibodies

**Figure 2:**
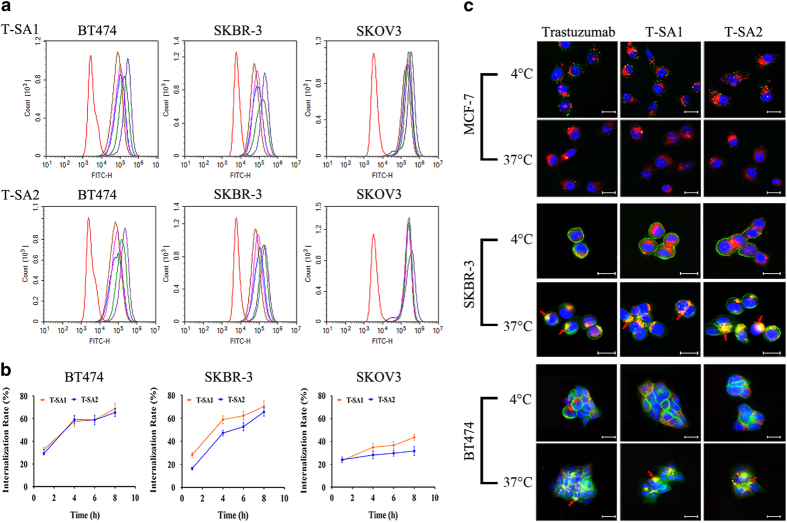
Internalization of T-SA1 and T-SA2 into HER2-positive cells detected by flow cytometry and immunofluorescence. (**a**) Flow cytometric analysis to evaluate the internalization efficiency of T-SA1 and T-SA2 in BT474, SKBR-3 and SKOV3; red: negative control group; purple: 0 h group; green: 1 h group; blue: 4 h group; pink: 6 h group; brown: 8 h group. (**b**) Uptake of scFv–HSA fusion antibodies in HER2-positive cells increased with time. (**c**) Internalization of trastuzumab, T-SA1 or T-SA2 into MCF-7, SKBR-3 and BT474 cells (scale bars, 12.5 μm). Cells were incubated with antibodies at 4 °C for 30 min. Unbinding antibodies were washed away. The experimental groups were incubated at 37 °C for 6 h.The green spots as shown were antibodies labeled with FITC. The blue spots were cell nuclei stained with 4,6-diamidino-2-phenylindole dihydrochloride. The red spots were lysosomes stained with Lyso-Tracker Red. The yellow to orange spots in the endochylema that were shown with the red arrows were the internalized antibodies co-localized with lysosomes.

To examine whether the scFv–HSA fusion antibodies could specifically internalize into HER2-positive tumor cells and study the internalization efficiency, the uptake of T-SA1 and T-SA2 in three kinds of HER2 high-expression cells (that is, BT474, SKBR-3 and SKOV3) was semi-quantified by flow cytometry assay. As shown in Figure 2a, along with the extension of incubation time, the magnitude of FITC peak shift decreased gradually, which represented an increasing number of scFv–HSA fusion antibodies were taken by cells. After incubating at 37 °C for 4 h, the internalization efficiency of both T-SA1 and T-SA2 reached ~50% in average in BT474 and SKBR-3 cells, and ~30% in SKOV3 cells (Figure 2b).

The distribution of T-SA1 and T-SA2 in HER2-positive cells SKBR-3 and BT474 as well as MCF-7 with low HER2 expression was detected via immunofluorescence. As shown in Figure 2c, the signal of fusion antibodies can be observed mainly on cellular membrane when incubated at exactly 4 °C. However, after internalization at 37 °C, the scFv–HSA fusion antibodies appeared as green spots in the endochylema of SKBR-3 and BT474 cells, but not in MCF-7 cells. By co-localizing with red staining of lysosome, we found that some green spots of antibodies and receptor complexes could overlap with the red spots of lysosomes and then displayed yellow-orange fluorescence. These results indicated that scFv–HSA fusion antibodies could enter lysosome degradation pathway after internalizing into cells.

### Preparation and characterization of scFv–HSA–DM1 conjugates

**Figure 3:**
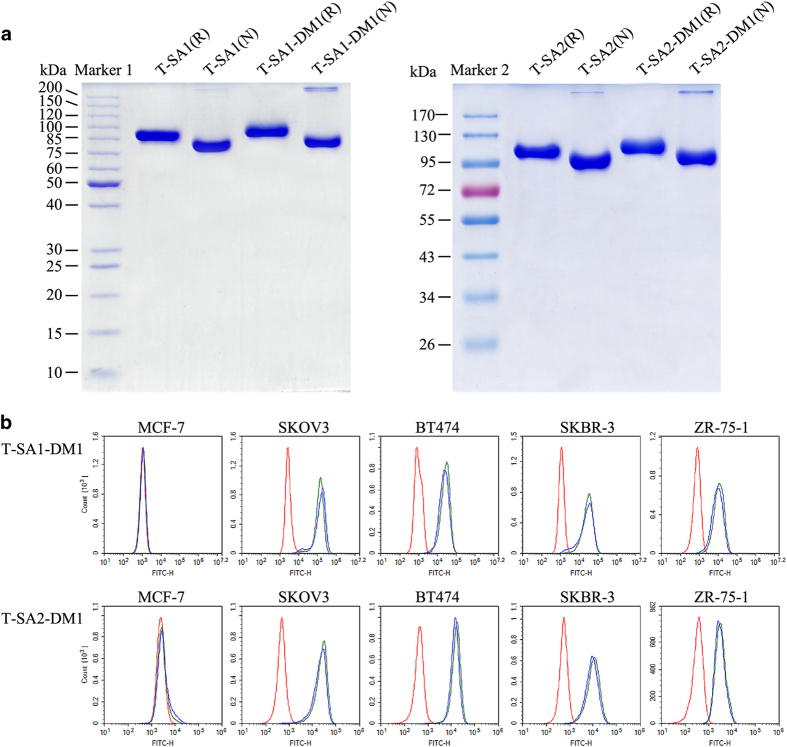
Characterization and comparison of scFv–HSA–DM1 conjugates. (**a**) SDS-PAGE analysis of scFv–HSA fusion antibodies and their conjugates. T-SA1, T-SA1–DM1, T-SA2 and T-SA2–DM1 loaded with reducing (R) and non-reducing loading buffer (N) were separated on 10% polyacrylamide gel. The images indicated that the anti-HER2 scFv conjugates showed similar electrophoretic behaviors with their unconjugated antibodies. Marker 1: PageRuler Unstained-Protein Ladder; marker 2: PageRuler Plus Prestained-Protein Ladder. (**b**) Binding activity of T-SA1–DM1 and T-SA2–DM1 to HER2-negative and -positive cells assessed by flow cytometry. T-SA1–DM1 and T-SA2–DM1 showed the same binding activity as their unconjugated antibodies. Red: negative control; green: T-SA1 or T-SA2; blue: T-SA1–DM1 or T-SA2–DM1.

Anti-HER2 scFv–HSA fusion antibodies and DM1–MCC were conjugated via the free amino group of lysine residue in neutral reaction buffer. T-SA1 and T-SA2 were exchanged into reaction buffer (50 mm potassium phosphate, 50 mm sodium chloride, 2 mm EDTA, pH 7.2) using AKTA Purifier 100 and Hitrap desalting column system (GE Healthcare, Pittsburgh, PA, USA). Dry powder of DM1–MCC was dissolved in dimethyl formamide and then mixed with scFv–HSA fusion antibodies in a molar ratio of 7.5:1 and volume ratio of 1:9 for the reaction at 25 °C for 12 h. After reaction, precipitate was removed by centrifuging at 17 000 *g* for 5 min and the free drug linkers were removed using Zeba Spin Desalting Columns (Thermo Fisher Scientific, Waltham, MA, USA). The DAR of scFv–HSA–DM1 conjugates was measured based on differential ultraviolet spectrophotometry. With higher molar ratio of DM1–MCC and antibodies or longer reaction time, high DAR could be obtained. Because of the hydrophobicity of DM1, scFv–HSA–DM1 conjugates become increasingly unstable with the increase of DAR. To maintain the stability of scFv–HSA–DM1 conjugates, the DAR of scFv–HSA–DM1 conjugations of each batch was kept between 3.2 and 3.5. As shown in Figure 3a, T-SA1–DM1 and T-SA2–DM1 showed similar electrophoretic behaviors with their unconjugated antibodies by SDS-PAGE analysis, except as lightly larger molecular weight because of the loaded DM1.

To examine whether conjugation with small molecules could affect the affinity and binding activity of scFv–HSA–DM1 conjugates, surface plasmon resonance analysis and flow cytometry assay were performed. According to surface plasmon resonance analysis, the affinity of these two conjugates was still in the same order of magnitude with antibodies before conjugation (T-SA1–DM1:T-SA1, 9.777×10^−11^ vs 5.910×10^−11^ m; T-SA2–DM1:T-SA2, 1.285×10^−10^ vs 1.300×10^−10^ m). As shown in Figure 3b, T-SA1–DM1 and T-SA2–DM1 showed the same binding activity as their unconjugated counterparts to HER2-positive cells (that is, SKOV3, BT474, SKBR-3 and ZR-75-1) and HER2-negative cell (MCF-7). These results indicated that conjugation of scFv–HSA fusion antibodies with appropriate small molecules did not affect the association and dissociation process of antibodies for HER2 receptor. Meanwhile, DM1–MCC might not conjugate in the key binding region with antigen of scFv–HSA fusion antibodies so that it did not affect the binding activity.

### *In vitro* cytotoxicity

The anti-proliferative activities of T-SA1–DM1 and T-SA2–DM1 on HER2-positive cells (SKOV3 and SKBR-3) and HER2-negative cells (MCF-7 and MDA-MB-231) were compared with T-SA1 and T-SA2 using Cell Counting Kit-8 assay, respectively. After 72 h exposure, the absorbance at 450 nm was measured and the IC_50_ values obtained were summarized in Table 1. ScFv–HSA–DM1 conjugates, similar to T-SA1 and T-SA2, showed little to no specific cytotoxicity on MCF-7 and MDA-MB-231 with low expression of HER2. The 72-h IC_50_ values of scFv–HSA–DM1 conjugates were >1200 and 750 nm respectively. In contrast, SKBR-3 cells with high level of HER2 expression were killed efficiently by both T-SA1–DM1 and T-SA2–DM1, with IC_50_ value of 1.05±0.03 and 1.10±0.09 nm. For another HER2-positive SKOV3 cells, T-SA1–DM1 and T-SA2–DM1 also showed outstanding inhibition and the IC_50_ values were 3.18±0.49 and 3.57±0.45 nm, respectively. However, the cell growth inhibition of scFv–HSA fusion antibodies on HER2-positive cells was not obvious even though their concentration reached 1200 nm. Compared with the unconjugated antibodies, anti-HER2 scFv–HSA–DM1 conjugates had stronger anti-proliferative activities on HER2-positive tumor cells. These data also indicated that high levels of cell surface HER2 were required for specific binding. Meanwhile, anti-proliferative activity was based on the internalization of conjugates and release of cytotoxic molecules DM1 by lysosomal degradation.

### *In vivo* antitumor activity

The antitumor activity of anti-HER2 scFv–HSA–DM1 conjugates, T-SA1–DM1 and T-SA2–DM1, was evaluated in HER2-positive human ovarian cancer xenograft models. Tolerability experiment was done prior to antitumor activity experiments in mice. Both the scFv–HSA–DM1 conjugates and unconjugated antibodies showed well tolerated at the dose of 30 mg kg^−1^ administrated via intravenous injection.

**Figure 4:**
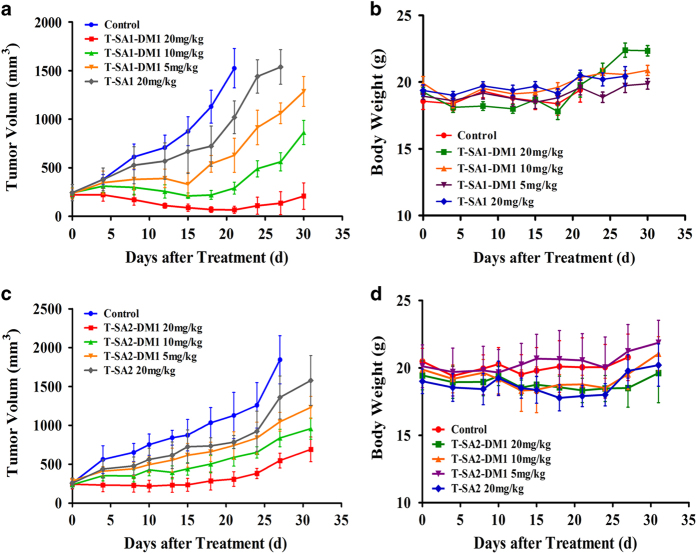
Antitumor activity of T-SA1–DM1 and T-SA2–DM1 against SKOV3 ovarian cancer xenografts. (**a**) Subcutaneous tumor-bearing mice were treated with storage buffer, T-SA1 (20 mg kg^−1^) and T-SA1–DM1 (5, 10 and 20 mg kg^−1^); (**c**) subcutaneous tumor-bearing mice were treated with storage buffer, T-SA2 (20 mg kg^−1^) and T-SA2–DM1 (5, 10 and 20 mg kg^−1^). Average tumor volumes were calculated and presented as growth curves. The body weight of nude mice did not change significantly during the treatment with T-SA1–DM1 (**b**) or T-SA2–DM1 (**d**).

Then tumor-bearing BALB/c nude mice were injected via tail vein with scFv–HSA–DM1 conjugates (5, 10 and 20 mg kg^−1^), unconjugated scFv–HSA fusion antibodies (20 mg kg^−1^) and control (storage buffer) on days 0, 4 and 8. As shown in Figures 4a and c, treatment with both scFv–HSA–DM1 conjugates and their unconjugated antibodies exhibited suppression of xenografts growth compared to controls. However, scFv–HSA–DM1 conjugates showed better antitumor activity than fusion antibodies even though at lower doses. Significant inhibition of tumor growth was observed with 20 mg kg^−1^ T-SA1–DM1- or T-SA2–DM1-treated groups, especially the T-SA1–DM1 treatment group, three out of six mice showed complete remission without regrowth. Tumor growth inhibition presented a dose-dependent effect. More than 1 week after drugs removal, the substantial tumors with non-complete remission in conjugate treatment group started to re-grow, but they showed slower growth rate compared to tumors in the control group and even in unconjugated antibody treatment group. Body weight was monitored twice a week and increased steadily in the treatment group, indicating that toxic effects were not obvious (Figures 4b and d). These findings indicated that scFv–HSA–DM1 conjugates, especially T-SA1–DM1, not only exhibited potent antitumor activity but also were well tolerated in the xenograft model.

### *In vivo* distribution of scFv–HSA fusion antibodies

**Figure 5:**
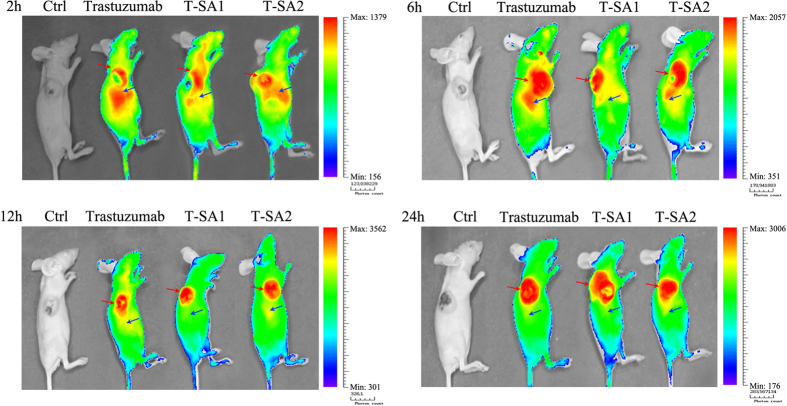
Distribution of trastuzumab, T-SA1 and T-SA2 *in vivo* was displayed by bioluminescence imaging system. Both T-SA1 and T-SA2 could target tumor tissues specifically within 2 h post injection and the maximum fluorescence intensity on the area of tumor was observed at 12 h post injection. The tumor tissue areas were shown with the red arrows. The liver areas were shown with the blue arrows.

To clarify the tumor-binding activity and distribution of T-SA1 and T-SA2 *in vivo*, trastuzumab, T-SA1 and T-SA2 labeled with Cy5.5 were administered via tail vein injection to tumor-bearing BALB/c nude mice and the images were collected by bioluminescence imaging system at 2, 6, 12 and 24 h post injection. As expected, both T-SA1 and T-SA2 groups were found under higher intensity in tumor tissues than normal tissues at 2 h post injection and to produce increasing signals over time until 12 h post injection as shown in Figure 5. In addition, among the three antibodies, T-SA1 showed the best tumor targeting property and the least accumulation in other organs. These phenomena might be due to the stronger penetrability of small molecular antibody. On the other hand, the capacity of HSA that is easy to accumulate in malignant and inflammatory tissues and to be utilized for tumor proliferation by their degradation products may play a role in the tumor accumulation in solid tumors.

**Figure 6:**
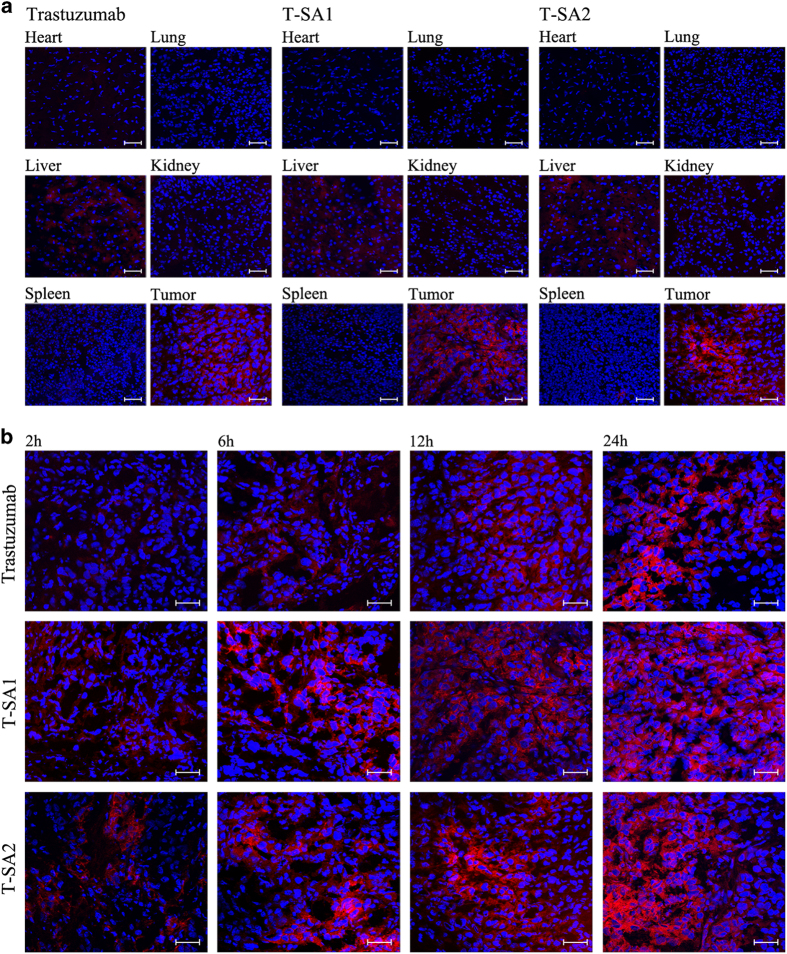
Immunofluorescence of tumor and primary organs samples after injection of trastuzumab, T-SA1 and T-SA2 (scale bars, 50 μm).Trastuzumab, T-SA1 and T-SA2 were detected in red fluorescence and cell nuclei were detected in blue fluorescence. (**a**) The same as trastuzumab, T-SA1 and T-SA2 distributed in tumor, liver and kidney tissues at 12 h post injection. Rather than appeared diffuse distribution in intercellular substance of liver and kidney tissues, antibodies accumulated in the cytoplasm in entire tumor tissues. (**b**) The fluorescence intensity of antibodies in tumor tissues increased with time. Among the three antibodies, T-SA1 had the widest distribution in tumor tissues.

To confirm precise distribution of T-SA1 and T-SA2 within the tumor tissues and main organs, tumors collected at each time point and organs (heart, liver, spleen, lung and kidney) collected at 12 h post injection were used for producing frozen sections. Frozen sections were observed under confocal microscope as shown in Figure 6. Trastuzumab, T-SA1 and T-SA2 were detected in red fluorescence and cell nuclei were detected in blue fluorescence. Frozen sections of different organs collected at 12 h post injection exhibited a little staining of fusion antibodies in liver and kidney tissues but not in other normal organs (Figure 6a). The red fluorescence of antibodies in the major metabolic organ liver and excretory organ kidney tissues appeared diffuse distribution in intercellular regions. However, in tumor tissues, antibodies mainly accumulated in the cytoplasm. These results verified that T-SA1 and T-SA2 had highly specific binding to HER2-positive tumor cells and efficient internalization *in vivo*.

As shown in Figure 6b, three different antibodies accumulated in the tumor areas gradually over time with the *in vivo* circulation. As we expected, T-SA2 showed wider distribution than the intact antibody trastuzumab in the tumor tissues. T-SA1, which was visible all over the tumors, appeared to accumulate best among three antibodies in the tumor tissues, and this might in part because of the strong tissues penetrability in solid tumor due to its small molecular size.

## Discussion

The release mechanism of cytotoxic drugs from ADCs is believed to occur after distribution in plasma, diffusion in tumor tissue, binding with cell surface antigen, internalization and degradation of the antibody component in lysosomes.^59^ Therefore, due to the limited tissue penetration of intact antibody, ADCs face enormous challenges in more effective treatment against solid tumors. To improve tumor tissue penetration and thus therapeutic efficacy, we developed a novel type of ADCs composed of anti-HER2 scFv–HSA fusion antibodies conjugated with the potent cytotoxic drug DM1 for HER2-positive cancer therapy.

The two anti-HER2 scFv–HSA fusion antibodies produced by transient expression system^60–63^ kept similar binding activity and high affinity as trastuzumab, and also could internalize into cells quickly as well as enter lysosome degradation pathway. On the basis of their characteristics, two ADCs complexes T-SA1–DM1 and T-SA2–DM1 were prepared, which were composed of scFv–HSA fusion antibodies conjugated with maytansine derivative DM1 on the sites of lysine residues via MCC linker. Published reports indicated that thioether (MCC) linker-containing ADCs not only internalized into tumor cells after binding to receptor on cell surface and undergo proteolytic degradation in the lysosome to release cytotoxic agents, but also maintained plasma stability more effectively.^57,59,64,65^ Clearly, high DAR results in ADCs with increased hydrophobicity and greater degree of destabilization. To ensure stability in pharmaceutically acceptable solvents, antigen-binding ability and antitumor activity of ADCs complexes, conjugation process was optimized to control the DAR of each batch in the range of 3.2–3.5.^66,67^ As expected, T-SA1–DM1 and T-SA2–DM1 had the same specific binding activity and affinity compared with their unconjugated antibodies, but showed significant inhibitory effect on the growth of HER2-positive cells and remarkable antitumor activity in mouse models. In addition, we also compared the *in vivo* antitumor activity of T-SA1–DM1 and T-SA2–DM1 with T-DM1 to evaluate the potential for the development of scFv–DM1 conjugates. Under the conditions of the same dose of injection (10 mg kg^−1^), T-SA1–DM1 showed similar inhibition of tumor growth with T-DM1 (data not shown). This result suggests that scFv–HSA–DM1 conjugates represent a promising antitumor drug candidate and warrant further study.

In general, therapeutic efficacy of ADCs is attributed to the combination of various factors. The mechanisms, including off-target effects, poor tissue penetration, low affinity, lack of internalization or drug resistance, may cause the disappointing treatment outcome.^4,68–70^ Such problems may be mitigated by designing conjugates that are in a protected form during the delivery and activated after binding to the target cells and internalization.^71^ Multiple reports further contend that antibody size and valence regulating *in vivo* tumor targeting and biodistribution are closely correlated with antitumor activity of ADCs. Therefore, ADCs consisting of intact antibodies that have relatively large molecular weight and limited tissue penetrability may be less effective in the treatment of solid tumors. Engineered antibodies featuring small size, high specificity with tumor tissues *in vivo*, and strong blood vessels and tissue penetrability, such as Fab, scFv and diabody, become potential guiding molecules of ADCs.^39,40,72^ However, the major obstacle on the clinical application of antibodies with small size lies in their short plasma half-life. Proteins with molecular weight above 55 kDa do not undergo first-pass elimination through the kidney, thus they have prolonged *in vivo* biodistribution.^73^ In this respect, modifications of antibody properties, like fusion with carrier proteins, PEGylation and linking with nanoparticles may strongly influence pharmacokinetics.^74^ In this study, anti-HER2 scFvs were fused with HSA as the guiding molecules of ADCs. The *in vivo* distribution experiment confirmed the corollary that T-SA1 with the smallest molecular weight possessed the best tumor targeting property and the least accumulation in other organs compared with trastuzumab even T-SA2, hence a highly specific delivery system. We suspected that the greater *in vivo* tumor targeting might be due to the higher penetrability of small molecular antibody and the characteristic of accumulation in malignant and inflammatory tissues of HSA,^75–78^ but the exact mechanism was unclear and in need of an accurate measure of pharmacokinetics and further verification.

Besides limited tissue penetration of ADCs, lacking efficient internalization of antibody component is another reason of disappointing treatment outcomes. Although our results proved that both T-SA1 and T-SA2 could internalize into the cells and enter lysosome degradation pathway, many studies considered that HER2 was resistant in internalization due to several mechanisms such as membrane protrusions and efficient recycle of internalized HER2 to membrane.^70,79–81^ It has been demonstrated that the stability of HER2 is associated with HSP90’s function, and HSP90 inhibitors played an important role in rapid downregulation of HER2 from the surface of cells.^82–84^ Meanwhile, studies by us have demonstrated that the internalization efficiency and lysosomal traffic of scFv–HSA fusion antibodies—HER2 complexes in HER2-positive tumor cells could be significantly improved by combining with HSP90 inhibitor 17-AAG (data not shown). This result is consistent with the view of Raja *et al.*^85^ It turned out that the potential combination of anti-HER2 scFv–HSA fusion antibody-based ADCs with an HSP90 inhibitor may provide a novel strategy to improve the therapeutic efficacy of cancer.

In addition, the valence of an antibody also plays a very important role in its tumor targeting capability and biodistribution. Although we speculated that the T-SA2–DM1 with bivalent antigen-binding sites should exhibit better antitumor activity, the *in vivo* study proved to be opposite.There are many reasons for this result, such as the molecular weight and size, the affinity, the stability of conformation and pharmacokinetics of antibody *in vivo*. Compared with an intact antibody, scFv presents a greater extent of aggregation. Moreover, an additional interaction occurring between V_H_ and V_L_ domains of two scFvs gives rise to form polymer more easily,^86,87^ so that scFv with bivalent antigen-binding sites is less stable. This might be the one reason for the slightly inferior affinity of T-SA2 compared with T-SA1. On the other hand, the arrangement order of V_H_ and V_L_ domains and the type of flexible linker are also important factors affecting the activity and stability of antibody.^88–91^ Therefore, the design of fusion antibodies needs to be further optimized in the future. On the basis of the results of *in vivo* distribution, the tumor tissue accumulation of T-SA1 was the most obvious. Therefore, the penetrability of antibody in tumor tissue is also the key factor influencing the therapeutic efficacy, and the related mechanism remains elusive and is worth further study. Furthermore, engineered antibodies have a variety of conformations, and our designed scFv–HSA fusion antibodies have only two. In the future, we will construct the preferred engineered antibodies through various screening of targeting ability, affinity, stability, penetrability and pharmacokinetic *in vivo*.

ADCs comprise three key components: an antibody conjugated to a high cytotoxicity compound via a linker. The antibody allows precise targeting of a drug to tumor cells. Beyond that, linker technology ensures the controllable and effective release of the drug in target cells rather than in plasma or normal tissues. Following the progress in ADCs development, a series of candidates may be screened out from the various combinations of three components to maximize drug delivery and to limit side effects at the same time. In conclusion, our designing strategy of ADCs based on scFv–HSA fusion antibody demonstrated the potential for applications in cancer therapy. Therefore, the structure optimization of antibody and screening of various linkers as well as agents deserve further exploration.

## Figures and Tables

**Table 1:** *In vitro* cytotoxicity of T-SA1–DM1 and T-SA2–DM1 (72 h exposure, IC_50_, nm)

*Material*	*MCF-7*	*MDA-MB-231*	*SKOV3*	*SKBR-3*
T-SA1	>1200	>1200	>1200	>1200
T-SA1–DM1	>1200	>750	3.18±0.49	1.05±0.03
T-SA2	>1200	>1200	>1200	>1200
T-SA2–DM1	>1200	>750	3.57±0.45	1.10±0.09

## References

[bib1] Feld J, Barta SK, Schinke C, Braunschweig I, Zhou Y, Verma A. Linked-in: design and efficacy of antibody drug conjugates in oncology. Oncotarget 2013; 4: 397–412. 23651630 10.18632/oncotarget.924 PMC3717303

[bib2] Casi G, Neri D. Antibody-drug conjugates: basic concepts, examples and future perspectives. J Control Release 2012; 161: 422–428. 22306430 10.1016/j.jconrel.2012.01.026

[bib3] Flemming A. Antibody engineering: fine-tuning antibody-drug conjugates. Nat Rev Drug Discov 2014; 13: 178–178. 24577396 10.1038/nrd4266

[bib4] Sievers EL, Senter PD. Antibody-drug conjugates in cancer therapy. Annu Rev Med 2013; 64: 15–29. 23043493 10.1146/annurev-med-050311-201823

[bib5] Crunkhorn S. Drug design: increasing stability of ADCs. Nat Rev Drug Discov 2014; 13: 812–812.25359376

[bib6] de Claro RA, McGinn K, Kwitkowski V, Bullock J, Khandelwal A, Habtemariam B et al. US Food and Drug Administration approval summary: brentuximab vedotin for the treatment of relapsed Hodgkin lymphoma or relapsed systemic anaplastic large-cell lymphoma. Clin Cancer Res 2012; 18: 5845–5849. 22962441 10.1158/1078-0432.CCR-12-1803

[bib7] Mathew J, Perez EA. Trastuzumab emtansine in human epidermal growth factor receptor 2-positive breast cancer: a review. Curr Opin Oncol 2011; 23: 594–600. 21986845 10.1097/CCO.0b013e32834b895c

[bib8] Shih C, Padhy L, Murray M, Weinberg RA. Transforming genes of carcinomas and neuroblastomas introduced into mouse fibroblasts. Nature 1981; 290: 261–264. 7207618 10.1038/290261a0

[bib9] Rubin I, Yarden Y. The basic biology of HER2. Ann Oncol 2001; 12(suppl 1): S3–S8. 10.1093/annonc/12.suppl_1.s3 11521719

[bib10] Nuti M, Bellati F, Visconti V, Napoletano C, Domenici L, Caccetta J et al. Immune effects of trastuzumab. J Cancer 2011; 2: 317–323. 21716848 10.7150/jca.2.317 PMC3119394

[bib11] Yarden Y, Sliwkowski MX. Untangling the ErbB signalling network. Nat Rev Mol Cell Biol 2001; 2: 127–137. 11252954 10.1038/35052073

[bib12] Jørgensen JT, Hersom M. HER2 as a prognostic marker in gastric cancer-a systematic analysis of data from the literature. J Cancer 2012; 3: 137–144. 22481979 10.7150/jca.4090 PMC3319979

[bib13] Verri E, Guglielmini P, Puntoni M, Perdelli L, Papadia A, Lorenzi P et al. HER2/neu oncoprotein overexpression in epithelial ovarian cancer: evaluation of its prevalence and prognostic significance. Oncology 2005; 68: 154–161. 16020953 10.1159/000086958

[bib14] Grob TJ, Kannengiesser I, Tsourlakis MC, Atanackovic D, Koenig AM, Vashist YK et al. Heterogeneity of ERBB2 amplification in adenocarcinoma, squamous cell carcinoma and large cell undifferentiated carcinoma of the lung. Mod Pathol 2012; 25: 1566–1573. 22899293 10.1038/modpathol.2012.125

[bib15] Iqbal N, Iqbal N. Human epidermal growth factor receptor 2 (HER2) in cancers: overexpression and therapeutic implications. Mol Biol Int 2014; 2014: 852748. 25276427 10.1155/2014/852748 PMC4170925

[bib16] Yoon HH, Shi Q, Sukov WR, Wiktor AE, Khan M, Sattler CA et al. Association of HER2/ErbB2 expression and gene amplification with pathologic features and prognosis in esophageal adenocarcinomas. Clin Cancer Res 2012; 18: 546–554. 22252257 10.1158/1078-0432.CCR-11-2272 PMC3261584

[bib17] Piccart-Gebhart MJ, Procter M, Leyland-Jones B, Goldhirsch A, Untch M, Smith I et al. Trastuzumab after adjuvant chemotherapy in HER2-positive breast cancer. N Engl J Med 2005; 353: 1659–1672. 16236737 10.1056/NEJMoa052306

[bib18] Tan AR, Swain SM. Ongoing adjuvant trials with trastuzumab in breast cancer. Semin Oncol 2003; 30(5 Suppl 16): 54–64. 10.1053/j.seminoncol.2003.08.008 14613027

[bib19] Bang Y-J, Van Cutsem E, Feyereislova A, Chung HC, Shen L, Sawaki A et al. Trastuzumab in combination with chemotherapy versus chemotherapy alone for treatment of HER2-positive advanced gastric or gastro-oesophageal junction cancer (ToGA): a phase 3, open-label, randomised controlled trial. Lancet 2010; 376: 687–697. 20728210 10.1016/S0140-6736(10)61121-X

[bib20] Hubalek M, Brantner C, Marth C. Role of pertuzumab in the treatment of HER2-positive breast cancer. Breast Cancer 2012; 4: 65–73. 24367194 10.2147/BCTT.S23560 PMC3846374

[bib21] Moya-Horno I, Cortés J. The expanding role of pertuzumab in the treatment of HER2-positive breast cancer. Breast Cancer 2015; 7: 125. 26056489 10.2147/BCTT.S61579 PMC4445592

[bib22] Boyraz B, Sendur MA, Aksoy S, Babacan T, Roach EC, Kizilarslanoglu MC et al. Trastuzumab emtansine (T-DM1) for HER2-positive breast cancer. Curr Med Res Opin 2013; 29: 405–414. 23402224 10.1185/03007995.2013.775113

[bib23] Lambert JM, Chari RV. Ado-trastuzumab emtansine (T-DM1): an antibody-drug conjugate (ADC) for HER2-positive breast cancer. J Med Chem 2014; 57: 6949–6964. 24967516 10.1021/jm500766w

[bib24] Geyer CE, Forster J, Lindquist D, Chan S, Romieu CG, Pienkowski T et al. Lapatinib plus capecitabine for HER2-positive advanced breast cancer. N Engl J Med 2006; 355: 2733–2743. 17192538 10.1056/NEJMoa064320

[bib25] Cetin B, Benekli M, Turker I, Koral L, Ulas A, Dane F et al. Lapatinib plus capecitabine for HER2-positive advanced breast cancer: a multicentre study of Anatolian Society of Medical Oncology (ASMO). J Chemother 2014; 26: 300–305. 24112786 10.1179/1973947813Y.0000000147

[bib26] Robidoux A, Tang G, Rastogi P, Geyer CE, Azar CA, Atkins JN et al. Lapatinib as a component of neoadjuvant therapy for HER2-positive operable breast cancer (NSABP protocol B-41): an open-label, randomised phase 3 trial. Lancet Oncol 2013; 14: 1183–1192. 24095300 10.1016/S1470-2045(13)70411-X

[bib27] Solca F, Dahl G, Zoephel A, Bader G, Sanderson M, Klein C et al. Target binding properties and cellular activity of afatinib (BIBW 2992), an irreversible ErbB family blocker. J Pharmacol Exp Ther 2012; 343: 342–350. 22888144 10.1124/jpet.112.197756

[bib28] Burris HA, Tibbitts J, Holden SN, Sliwkowski MX, Phillips GDL. Trastuzumab emtansine (T-DM1): a novel agent for targeting HER2+ breast cancer. Clin Breast Cancer 2011; 11: 275–282. 21729661 10.1016/j.clbc.2011.03.018

[bib29] Burris HA, Rugo HS, Vukelja SJ, Vogel CL, Borson RA, Limentani S et al. Phase II study of the antibody drug conjugate trastuzumab-DM1 for the treatment of human epidermal growth factor receptor 2 (HER2)-positive breast cancer after prior HER2-directed therapy. J Clin Oncol 2011; 29: 398–405. 21172893 10.1200/JCO.2010.29.5865

[bib30] Krop IE, LoRusso P, Miller KD, Modi S, Yardley D, Rodriguez G et al. A phase II study of trastuzumab emtansine in patients with human epidermal growth factor receptor 2-positive metastatic breast cancer who were previously treated with trastuzumab, lapatinib, an anthracycline, a taxane, and capecitabine. J Clin Oncol 2012; 30: 3234–3241. 22649126 10.1200/JCO.2011.40.5902

[bib31] Krop IE, Kim S-B, González-Martín A, LoRusso PM, Ferrero J-M, Smitt M et al. Trastuzumab emtansine versus treatment of physician's choice for pretreated HER2-positive advanced breast cancer (TH3RESA): a randomised, open-label, phase 3 trial. Lancet Oncol 2014; 15: 689–699. 24793816 10.1016/S1470-2045(14)70178-0

[bib32] Kupchan SM, Komoda Y, Court W, Thomas G, Smith R, Karim A et al. Tumor inhibitors. LXXIII. Maytansine, a novel antileukemic ansa macrolide from *Maytenus ovatus*. J Am Chem Soc 1972; 94: 1354–1356. 5062169 10.1021/ja00759a054

[bib33] Verma S, Miles D, Gianni L, Krop IE, Welslau M, Baselga J et al. Trastuzumab emtansine for HER2-positive advanced breast cancer. N Engl J Med 2012; 367: 1783–1791. 23020162 10.1056/NEJMoa1209124 PMC5125250

[bib34] Foyil KV, Bartlett NL. *Brentuximab vedotin* for the treatment of CD30+ lymphomas. Immunotherapy 2011; 3: 475–485. 21463188 10.2217/imt.11.15

[bib35] Younes A, Gopal AK, Smith SE, Ansell SM, Rosenblatt JD, Savage KJ et al. Results of a pivotal phase II study of brentuximab vedotin for patients with relapsed or refractory Hodgkin's lymphoma. J Clin Oncol 2012; 30: 2183–2189. 22454421 10.1200/JCO.2011.38.0410 PMC3646316

[bib36] Pro B, Advani R, Brice P, Bartlett NL, Rosenblatt JD, Illidge T et al. Brentuximab vedotin (SGN-35) in patients with relapsed or refractory systemic anaplastic large-cell lymphoma: results of a phase II study. J Clin Oncol 2012; 30: 2190–2196. 22614995 10.1200/JCO.2011.38.0402

[bib37] Moskowitz CH, Nademanee A, Masszi T, Agura E, Holowiecki J, Abidi MH et al. Brentuximab vedotin as consolidation therapy after autologous stem-cell transplantation in patients with Hodgkin's lymphoma at risk of relapse or progression (AETHERA): a randomised, double-blind, placebo-controlled, phase 3 trial. Lancet 2015; 385: 1853–1862. 25796459 10.1016/S0140-6736(15)60165-9

[bib38] Azemar M, Schmidt M, Arlt F, Kennel P, Brandt B, Papadimitriou A et al. Recombinant antibody toxins specific for ErbB2 and EGF receptor inhibit the *in vitro* growth of human head and neck cancer cells and cause rapid tumor regression *in vivo*. Int J Cancer 2000; 86: 269–275. 10738256 10.1002/(sici)1097-0215(20000415)86:2<269::aid-ijc18>3.0.co;2-8

[bib39] Cao Y, Marks JD, Huang Q, Rudnick SI, Xiong C, Hittelman WN et al. Single-chain antibody-based immunotoxins targeting Her2/neu: design optimization and impact of affinity on antitumor efficacy and off-target toxicity. Mol Cancer Ther 2012; 11: 143–153. 22090420 10.1158/1535-7163.MCT-11-0519

[bib40] Cao Y, Marks JD, Marks JW, Cheung LH, Kim S, Rosenblum MG. Construction and characterization of novel, recombinant immunotoxins targeting the Her2/neu oncogene product: *in vitro* and *in vivo* studies. Cancer Res 2009; 69: 8987–8995. 19934334 10.1158/0008-5472.CAN-09-2693

[bib41] Cao Y, Marks JW, Liu Z, Cheung LH, Hittelman WN, Rosenblum MG. Design optimization and characterization of Her2/neu-targeted immunotoxins: comparative *in vitro* and *in vivo* efficacy studies. Oncogene 2014; 33: 429–439. 23376850 10.1038/onc.2012.612 PMC4527163

[bib42] Arbabi-Ghahroudi M, Tanha J, MacKenzie R. Prokaryotic expression of antibodies. Cancer Metastasis Rev 2005; 24: 501–519. 16408159 10.1007/s10555-005-6193-1

[bib43] Furukawa M, Tanaka R, Chuang VTG, Ishima Y, Taguchi K, Watanabe H et al. Human serum albumin-thioredoxin fusion protein with long blood retention property is effective in suppressing lung injury. J Control Release 2011; 154: 189–195. 21620911 10.1016/j.jconrel.2011.05.013

[bib44] Hoppmann S, Miao Z, Liu S, Liu H, Ren G, Bao A et al. Radiolabeled affibody-albumin bioconjugates for HER2-positive cancer targeting. Bioconjug Chem 2011; 22: 413–421. 21299201 10.1021/bc100432h PMC3059402

[bib45] Yao Y, Su X, Xie Y, Wang Y, Kang T, Gou L et al. Synthesis, characterization, and antitumor evaluation of the albumin-SN38 conjugate. Anti-cancer drugs 2013; 24: 270–277. 23233044 10.1097/CAD.0b013e32835c3543

[bib46] Kratz F, Warnecke A, Scheuermann K, Stockmar C, Schwab J, Lazar P et al. Probing the cysteine-34 position of endogenous serum albumin with thiol-binding doxorubicin derivatives. Improved efficacy of an acid-sensitive doxorubicin derivative with specific albumin-binding properties compared to that of the parent compound. J Med Chem 2002; 45: 5523–5533. 12459020 10.1021/jm020276c

[bib47] Kratz F, Mansour A, Soltau J, Warnecke A, Fichtner I, Unger C et al. Development of albumin-binding doxorubicin prodrugs that are cleaved by prostate-specific antigen. Arch Pharm 2005; 338: 462–472. 10.1002/ardp.200500130 16211657

[bib48] Kratz F. Albumin as a drug carrier: design of prodrugs, drug conjugates and nanoparticles. J Control Release 2008; 132: 171–183. 18582981 10.1016/j.jconrel.2008.05.010

[bib49] Cai Z, Fu T, Nagai Y, Lam L, Yee M, Zhu Z et al. scFv-based “grababody” as a general strategy to improve recruitment of immune effector cells to antibody-targeted tumors. Cancer Res 2013; 73: 2619–2627. 23396586 10.1158/0008-5472.CAN-12-3920 PMC3630244

[bib50] Longva KE, Pedersen NM, Haslekås C, Stang E, Madshus IH. Herceptin-induced inhibition of ErbB2 signaling involves reduced phosphorylation of Akt but not endocytic down-regulation of ErbB2. Int J Cancer 2005; 116: 359–367. 15800944 10.1002/ijc.21015

[bib51] Raja SM, Clubb RJ, Bhattacharyya M, Dimri M, Cheng H, Pan W et al. A combination of Trastuzumab and 17-AAG induces enhanced ubiquitinylation and lysosomal pathway-dependent ErbB2 degradation and cytotoxicity in ErbB2-overexpressing breast cancer cells. Cancer Biol Ther 2008; 7: 1630–1640. 18769124 10.4161/cbt.7.10.6585 PMC2727620

[bib52] Hashizume T, Fukuda T, Nagaoka T, Tada H, Yamada H, Watanabe K et al. Cell type dependent endocytic internalization of ErbB2 with an artificial peptide ligand that binds to ErbB2. Cell Biol Int 2008; 32: 814–826. 18442934 10.1016/j.cellbi.2008.03.012

[bib53] Belimezi MM, Papanastassiou D, Merkouri E, Baxevanis CN, Mamalaki A. Growth inhibition of breast cancer cell lines overexpressing Her2/neu by a novel internalized fully human Fab antibody fragment. Cancer Immunol Immunother 2006; 55: 1091–1099. 16311733 10.1007/s00262-005-0100-z PMC11030719

[bib54] Zhu X, Bidlingmaier S, Hashizume R, James CD, Berger MS, Liu B. Identification of internalizing human single-chain antibodies targeting brain tumor sphere cells. Mol Cancer Ther 2010; 9: 2131–2141. 20587664 10.1158/1535-7163.MCT-09-1059 PMC2944778

[bib55] Sommaruga S, Lombardi A, Salvadè A, Mazzucchelli S, Corsi F, Galeffi P et al. Highly efficient production of anti-HER2 scFv antibody variant for targeting breast cancer cells. Appl Microbiol Biotechnol 2011; 91: 613–621. 21538107 10.1007/s00253-011-3306-3

[bib56] Kramer-Marek G, Kiesewetter DO, Capala J. Changes in HER2 expression in breast cancer xenografts after therapy can be quantified using PET and 18 F-labeled affibody molecules. J Nucl Med 2009; 50: 1131–1139. 19525458 10.2967/jnumed.108.057695 PMC2787241

[bib57] Phillips GDL, Li G, Dugger DL, Crocker LM, Parsons KL, Mai E et al. Targeting HER2-positive breast cancer with trastuzumab-DM1, an antibody-cytotoxic drug conjugate. Cancer Res 2008; 68: 9280–9290. 19010901 10.1158/0008-5472.CAN-08-1776

[bib58] Zhang M, Qiu Z, Li Y, Yang Y, Zhang Q, Xiang Q et al. Construction and characterization of a recombinant human beta defensin 2 fusion protein targeting the epidermal growth factor receptor: *in vitro* study. Appl Microbiol Biotechnol 2013; 97: 3913–3923. 22903275 10.1007/s00253-012-4257-z

[bib59] Ducry L, Stump B. Antibody-drug conjugates: linking cytotoxic payloads to monoclonal antibodies. Bioconjug Chem 2009; 21: 5–13. 10.1021/bc9002019 19769391

[bib60] Backliwal G, Hildinger M, Hasija V, Wurm FM. High-density transfection with HEK-293 cells allows doubling of transient titers and removes need for a priori DNA complex formation with PEI. Biotechnol Bioeng 2008; 99: 721–727. 17680657 10.1002/bit.21596

[bib61] Baldi L, Hacker DL, Adam M, Wurm FM. Recombinant protein production by large-scale transient gene expression in mammalian cells: state of the art and future perspectives. Biotechnol Lett 2007; 29: 677–684. 17235486 10.1007/s10529-006-9297-y

[bib62] Cheng L, Sun X, Yi X, Zhang Y. Large-scale plasmid preparation for transient gene expression. Biotechnol Lett 2011; 33: 1559–1564. 21476094 10.1007/s10529-011-0612-x

[bib63] Fliedl L, Kaisermayer C. Transient gene expression in HEK293 and vero cells immobilised on microcarriers. J Biotechnol 2011; 153: 15–21. 21356254 10.1016/j.jbiotec.2011.02.007

[bib64] Erickson HK, Park PU, Widdison WC, Kovtun YV, Garrett LM, Hoffman K et al. Antibody-maytansinoid conjugates are activated in targeted cancer cells by lysosomal degradation and linker-dependent intracellular processing. Cancer Res 2006; 66: 4426–4433. 16618769 10.1158/0008-5472.CAN-05-4489

[bib65] Xie H, Audette C, Hoffee M, Lambert JM, Blättler WA. Pharmacokinetics and biodistribution of the antitumor immunoconjugate, cantuzumab mertansine (huC242-DM1), and its two components in mice. J Pharmacol Exp Ther 2004; 308: 1073–1082. 14634038 10.1124/jpet.103.060533

[bib66] Hamblett KJ, Senter PD, Chace DF, Sun MM, Lenox J, Cerveny CG et al. Effects of drug loading on the antitumor activity of a monoclonal antibody drug conjugate. Clin Cancer Res 2004; 10: 7063–7070. 15501986 10.1158/1078-0432.CCR-04-0789

[bib67] Stephan J-P, Chan P, Lee C, Nelson C, Elliott JM, Bechtel C et al. Anti-CD22-MCC-DM1 and MC-MMAF conjugates: impact of assay format on pharmacokinetic parameters determination. Bioconjug Chem 2008; 19: 1673–1683. 18637680 10.1021/bc800059t

[bib68] Hayes DF, Yamauchi H, Broadwater G, Cirrincione CT, Rodrigue SP, Berry DA et al. Circulating HER-2/erbB-2/c-neu (HER-2) extracellular domain as a prognostic factor in patients with metastatic breast cancer cancer and leukemia group B study 8662. Clin Cancer Res 2001; 7: 2703–2711. 11555582

[bib69] Molina MA, Sáez R, Ramsey EE, Garcia-Barchino M-J, Rojo F, Evans AJ et al. NH(2)-terminal truncated HER-2 protein but not full-length receptor is associated with nodal metastasis in human breast cancer. Clin Cancer Res 2002; 8: 347–353. 11839648

[bib70] Austin CD, De Mazière AM, Pisacane PI, van Dijk SM, Eigenbrot C, Sliwkowski MX et al. Endocytosis and sorting of ErbB2 and the site of action of cancer therapeutics trastuzumab and geldanamycin. Mol Biol Cell 2004; 15: 5268–5282. 15385631 10.1091/mbc.E04-07-0591 PMC532009

[bib71] Huang C, Liu Y, Rokita SE. Targeting duplex DNA with the reversible reactivity of quinone methides. Signal Transduct Target Ther 2016; 1: 16009. 28458944 10.1038/sigtrans.2016.9 PMC5407369

[bib72] Smaglo BG, Aldeghaither D, Weiner LM. The development of immunoconjugates for targeted cancer therapy. Nat Rev Clin Oncol 2014; 11: 637–648. 25265912 10.1038/nrclinonc.2014.159 PMC4700536

[bib73] Yokota T, Milenic DE, Whitlow M, Wood JF, Hubert SL, Schlom J. Microautoradiographic analysis of the normal organ distribution of radioiodinated single-chain Fv and other immunoglobulin forms. Cancer Res 1993; 53: 3776–3783. 8339291

[bib74] Pasquetto MV, Vecchia L, Covini D, Digilio R, Scotti C. Targeted drug delivery using immunoconjugates: principles and applications. J Immunother 2011; 34: 611–628. 21989410 10.1097/CJI.0b013e318234ecf5

[bib75] Liu F, Mu J, Xing B. Recent advances on the development of pharmacotherapeutic agents on the basis of human serum albumin. Curr Pharm Des 2015; 21: 1866–1888. 25732552 10.2174/1381612821666150302115411

[bib76] Rogers B, Dong D, Li Z, Li Z. Recombinant human serum albumin fusion proteins and novel applications in drug delivery and therapy. Curr Pharm Des 2015; 21: 1899–1907. 25732550 10.2174/1381612821666150302120047

[bib77] Qi W-W, Yu H-Y, Guo H, Lou J, Wang Z-M, Liu P et al. Doxorubicin-loaded glycyrrhetinic acid modified recombinant human serum albumin nanoparticles for targeting liver tumor chemotherapy. Mol Pharm 2015; 12: 675–683. 25584860 10.1021/mp500394v

[bib78] Müller D, Karle A, Meißburger B, Höfig I, Stork R, Kontermann RE. Improved pharmacokinetics of recombinant bispecific antibody molecules by fusion to human serum albumin. J Biol Chem 2007; 282: 12650–12660. 17347147 10.1074/jbc.M700820200

[bib79] Hommelgaard AM, Lerdrup M, van Deurs B. Association with membrane protrusions makes ErbB2 an internalization-resistant receptor. Mol Biol Cell 2004; 15: 1557–1567. 14742716 10.1091/mbc.E03-08-0596 PMC379255

[bib80] Rudnick SI, Lou J, Shaller CC, Tang Y, Klein-Szanto AJ, Weiner LM et al. Influence of affinity and antigen internalization on the uptake and penetration of anti-HER2 antibodies in solid tumors. Cancer Res 2011; 71: 2250–2259. 21406401 10.1158/0008-5472.CAN-10-2277 PMC3077882

[bib81] Bertelsen V, Stang E. The mysterious ways of ErbB2/HER2 trafficking. Membranes 2014; 4: 424–446. 25102001 10.3390/membranes4030424 PMC4194043

[bib82] Citri A, Kochupurakkal BS, Yarden Y. The achilles heel of ErbB-2/HER2: regulation by the Hsp90 chaperone machine and potential for pharmacological intervention. Cell Cycle 2004; 3: 50–59. 14657666

[bib83] Lerdrup M, Hommelgaard AM, Grandal M, van Deurs B. Geldanamycin stimulates internalization of ErbB2 in a proteasome-dependent way. J Cell Sci 2006; 119: 85–95. 16352662 10.1242/jcs.02707

[bib84] Pedersen NM, Madshus IH, Haslekås C, Stang E. Geldanamycin-induced down-regulation of ErbB2 from the plasma membrane is clathrin dependent but proteasomal activity independent. Mol Cancer Res 2008; 6: 491–500. 18337455 10.1158/1541-7786.MCR-07-0191

[bib85] Raja SM, Desale SS, Mohapatra B, Luan H, Soni K, Zhang J et al. Marked enhancement of lysosomal targeting and efficacy of ErbB2-targeted drug delivery by HSP90 inhibition. Oncotarget 2016; 7: 10522–10535. 26859680 10.18632/oncotarget.7231 PMC4891137

[bib86] Perisic O, Webb PA, Holliger P, Winter G, Williams RL. Crystal structure of a diabody, a bivalent antibody fragment. Structure 1994; 2: 1217–1226. 7704531 10.1016/s0969-2126(94)00123-5

[bib87] Arndt MA, Krauss J, Rybak SM. Antigen binding and stability properties of non-covalently linked anti-CD22 single-chain Fv dimers. FEBS Lett 2004; 578: 257–261. 15589829 10.1016/j.febslet.2004.11.011

[bib88] Huston JS, Mudgett-Hunter M, Tai MS, McCartney J, Warren F, Haber E et al. Protein engineering of single-chain Fv analogs and fusion proteins. Methods Enzymol 1991; 203: 46–88. 1762568 10.1016/0076-6879(91)03005-2

[bib89] Whitlow M, Bell BA, Feng SL, Filpula D, Hardman KD, Hubert SL et al. An improved linker for single-chain Fv with reduced aggregation andenhanced proteolytic stability. Protein Eng 1993; 6: 989–995. 8309948 10.1093/protein/6.8.989

[bib90] Lu D, Jimenez X, Witte L, Zhu Z. The effect of variable domainorientation and arrangement on the antigen-binding activity of arecombinant human bispecific diabody. Biochem Biophys Res Commun 2004; 318: 507–513. 15120630 10.1016/j.bbrc.2004.04.060

[bib91] Sheikholvaezin A, Sandstrom P, Eriksson D, Norgren N, Riklund K, Stigbrand T. Optimizing the generation of recombinant single-chainantibodies against placental alkaline phosphatase. Hybridoma 2006; 25: 181–192. 16934014 10.1089/hyb.2006.25.181

